# Impact of Low-Cost Point-of-Use Water Treatment Technologies on Enteric Infections and Growth among Children in Limpopo, South Africa

**DOI:** 10.4269/ajtmh.20-0228

**Published:** 2020-08-24

**Authors:** Courtney L. Hill, Kelly McCain, Mzwakhe E. Nyathi, Joshua N. Edokpayi, David M. Kahler, Darwin J. Operario, David D. J. Taylor, Natasha C. Wright, James A. Smith, Richard L. Guerrant, Amidou Samie, Rebecca A. Dillingham, Pascal O. Bessong, Elizabeth T. Rogawski McQuade

**Affiliations:** 1 Department of Engineering Systems and Environment, University of Virginia, Charlottesville, Virginia;; 2 Department of Epidemiology, Rollins School of Public Health, Emory University, Atlanta, Georgia;; 3 Department of Animal Science, University of Venda, Thohoyandou, South Africa;; 4 Department of Hydrology and Water Resources Mining and Environmental Geology, University of Venda, Thohoyandou, South Africa;; 5 Center for Environmental Research and Education, Duquesne University, Pittsburgh, Pennsylvania;; 6 Division of Infectious Diseases and International Health, University of Virginia, Charlottesville, Virginia;; 7 Department of Civil and Mineral Engineering, University of Toronto, Toronto, Canada;; 8 Department of Mechanical Engineering, University of Minnesota Twin Cities, Minneapolis, Minnesota;; 9 Department of Microbiology, University of Venda, Thohoyandou, South Africa;; 10 Department of Public Health Sciences, University of Virginia, Charlottesville, Virginia

## Abstract

Enteric infections early in life have been associated with poor linear growth among children in low-resource settings. Point-of-use water treatment technologies provide effective and low-cost solutions to reduce exposure to enteropathogens from drinking water, but it is unknown whether the use of these technologies translates to improvements in child growth. We conducted a community-based randomized controlled trial of two water treatment technologies to estimate their effects on child growth in Limpopo, South Africa. We randomized 404 households with a child younger than 3 years to receive a silver-impregnated ceramic water filter, a silver-impregnated ceramic tablet, a safe-storage water container alone, or no intervention, and these households were followed up quarterly for 2 years. We estimated the effects of the interventions on linear and ponderal growth, enteric infections assessed by quantitative molecular diagnostics, and diarrhea prevalence. The silver-impregnated ceramic water filters and tablets consistently achieved approximately 1.2 and 3 log reductions, respectively, in total coliform bacteria in drinking water samples. However, the filters and tablets were not associated with differences in height (height-for-age *z*-score differences compared with no intervention: 0.06, 95% CI: −0.29, 0.40, and 0.00, 95% CI: −0.35, 0.35, respectively). There were also no effects of the interventions on weight, diarrhea prevalence, or enteric infections. Despite their effectiveness in treating drinking water, the use of the silver-impregnated ceramic water filters and tablets did not reduce enteric infections or improve child growth. More transformative water, sanitation, and hygiene interventions that better prevent enteric infections are likely needed to improve long-term child growth outcomes.

## INTRODUCTION

In low-resource settings, lack of safe water has long-term detrimental consequences for child health and development. Exposure to enteropathogens in contaminated drinking water not only causes enteric infections and diarrhea but also may contribute to environmental enteropathy, an inflammatory condition of the gut associated with increased intestinal permeability, impaired gut immune function, and malabsorption.^1^ The cumulative impact of this exposure has been associated with poor linear growth and stunting,^2,3^ which affects approximately 162 million^4^ or 27% of children^5^ younger than 5 years globally. Stunting early in life has been associated with cognitive impairment, poor school performance, low adult economic productivity, and increased risk of chronic disease later in life.^6,7^

In 2012, the WHO adopted a resolution with a global target to reduce the number of stunted children younger than 5 years by 40% by 2025.^6^ Interventions to improve water, sanitation, and hygiene (WASH) were identified as an essential component of efforts to reach this goal.^3^ The first 2 years of life are a critical period and provide the optimal window to prevent child stunting.^6,7^ However, until the recently published WASH Benefits^8,9^ and The Sanitation, Hygiene, Infant Nutrition Efficacy (SHINE)^10^ trials, few WASH intervention trials examined child growth as a primary outcome or assessed enteric infections, an important marker of the interruption of fecal–oral microbial transmission. Earlier studies have focused mainly on caregiver-reported diarrhea, which is subject to recall bias.^11^ Interventions that improve access to safe water have the potential to make an impact on child growth and enteric infections, but have not been well studied toward this aim.

Strategies to increase access to clean water are challenged by recontamination between water collection and use.^12^ Point-of-use (POU) water treatment technologies treat drinking water in the household before it is consumed, eliminating the risk of contamination both at the source and during transport to the household.^13,14^ Sustainable, socially acceptable, and low-cost interventions with these technologies have the potential to improve the microbial quality of household water, reduce pathogen exposure to children, and prevent diarrheal diseases.^15–18^

Our research team has participated in the development and implementation of two POU water technologies that have demonstrated technical efficacy, sustainability, and social acceptance in low-resource communities. The first, a silver-impregnated ceramic water filter, is a well-developed, tested, and widely used device.^19,20^ In addition to mechanically removing turbidity and pathogens, the filter is treated with silver to kill live pathogens that pass through the filter and to provide residual disinfectant to reduce risk of recontamination after treatment. Silver-impregnated ceramic water filters can be more cost effective, exhibit lower environmental impacts (e.g., energy consumption and global warming potential), and show more potential for quality of life improvement than centralized water treatment and distribution systems.^21^

The second technology is a silver-impregnated ceramic tablet (MadiDrop™, Silivhere Technologies, Inc., Charlottesville, VA).^22,23^ When the ceramic tablet is placed in a household water storage container, silver diffuses through the porous ceramic into the water at a release rate that is effective for continual disinfection of waterborne pathogens while remaining below the silver drinking water standard. The tablet is effective for daily treatment of 10 L over 24 hours for at least 6 months. It can be used alone or in combination with a water filter, which may be most effective in situations with high water turbidity.

Both technologies have proven to be highly effective in treating water, showing up to a 3.2 log reduction in total coliform bacteria during field studies in South Africa and Tanzania.^23,24^ Ceramic water filters also reduced diarrheal rates among HIV-positive individuals in Limpopo, South Africa, by 79%.^15^ However, it is unknown whether the use of these technologies can translate to improvements in child health outcomes, particularly linear growth. We conducted a community-based randomized controlled intervention trial to estimate the effectiveness of the silver-impregnated ceramic water filter and the silver-impregnated ceramic tablet to improve child growth in Limpopo, South Africa.

## METHODS

### Participants.

Eligible households were identified in rural villages in the Dzimauli community in Limpopo, South Africa, between June and November 2016. Field-workers took a census of the villages by visiting each house to determine whether the household met the inclusion criteria for the study: mother was in the third trimester of pregnancy or there was at least one child younger than 3 years in the household. Households were excluded if they had chlorinated water piped into the home or routinely delivered (via truck or diversion) to a permanent, engineered system that stored the water within the property, they used a ceramic filter or other commercial water treatment system (including a permanent, engineered system that treats the water through filtration and/or chlorination), they had plans to move outside the community in the next 6 months, the child’s caregiver was younger than 16 years or unable to give consent, or the youngest child aged less than 3 years was seriously ill (had a severe disease requiring prolonged hospitalization or a severe or chronic condition diagnosed by medical doctor, e.g., neonatal disease, renal disease, chronic heart failure, liver disease, cystic fibrosis, and congenital conditions). If eligible, caregivers were asked to participate, and written informed consent was obtained.

The study protocol was approved by the University of Virginia Institutional Review Board for Health Sciences Research (18662) and the University of Venda Research Ethics Committee (SMNS/15/MBY/27/0502). This study was registered at clinicaltrials.gov (NCT03012048).

### Interventions.

Enrolled households were randomized to receive the following: 1) a silver-impregnated ceramic water filter inside a 20-L safe storage container (covered plastic bucket with a spigot) with two silver-impregnated ceramic tablets in the lower reservoir, 2) two silver-impregnated ceramic tablets inside a 20-L safe storage container, 3) a safe storage container alone, or 4) no intervention. The safe storage container alone arm was included to estimate the effectiveness of a designated, covered container for clean drinking water that limits recontamination from dirty hands. Randomization was stratified by the age (0–11 months, 12–23 months, and 23–35 months) of the youngest child in the household aged less than 3 years (henceforth, primary study child) and was conducted in blocks of 4. Details for preparation of each intervention and instructions given to participants have been described.^25–29^

Because the silver-impregnated ceramic tablet was designed to treat 10 L of water, two tablets were initially chosen to treat water in the 20-L container. During follow-up, the silver concentration in some treated water samples exceeded the WHO drinking water recommended guideline value of 100 µg/L silver.^30^ In coordination with our data safety and monitoring committee, we made several modifications to the interventions to ensure silver concentrations were less than the designated limit. In October 2016, the two silver-impregnated ceramic tablets in the lower reservoir of the storage container were removed from the filter group (1 above), and one silver-impregnated ceramic tablet was removed from the tablet-only group (2 above). In May 2017, the silver-impregnated ceramic tablets were replaced with those that were manufactured with 50% of the original amount of silver. In August 2017, all silver-impregnated ceramic tablets were replaced with silver-impregnated ceramic filters. In December 2017, all silver-impregnated ceramic filters were replaced with ceramic filters that did not contain silver.^25^ Based on the interventions in place during most of the follow-up, we refer to the interventions for the remainder of the article as 1) a silver-impregnated ceramic water filter, 2) a silver-impregnated ceramic tablet, 3) a safe storage container, and 4) no intervention.

### Power calculations.

We aimed to enroll at least 400 households (approximately 100 per randomized arm). Assuming a 20% dropout rate, we expected 320 households (80 per randomized arm) to complete follow-up. Assuming a baseline mean height-for-age *z*-score (HAZ) of −1.67 (the mean HAZ at 2 years in the the Etiology, Risk Factors, and Interactions of Enteric Infections and Malnutrition and the Consequences for Child Health and Development study [MAL-ED] South Africa study),^2^ we would have 80% power to detect a 27% difference in ΔHAZ (0.45 *z*-score difference) from baseline to the end of follow-up in pairwise comparisons between intervention and control groups (alpha level of 0.05 and two-sided test). We would have 80% power to detect a 19% difference in ΔHAZ (0.31 *z*-score difference) in the comparison that combines the two intervention and two control arms.

### Data collection.

A baseline visit was conducted to install the interventions and train caregivers on intervention use and maintenance. Field-workers encouraged participants to use the interventions for all drinking water in the household. A baseline questionnaire was conducted concerning demographics, socioeconomic status, water sources, sanitation and hygiene practices, and 7-day diarrhea prevalence in the primary study child. Height and weight were measured, and a stool sample was collected from the primary study child. Length was measured in children younger than 24 months of age using a recumbent measuring board (Seca, Hamburg, Germany), and height was measured in children older than 24 months using a stadiometer. For each measurement, readings were taken twice and the average recorded to the nearest 0.1 cm. Weight was measured with a digital scale (Seca), also taken twice, and the average reported to the nearest 10 g. For quality assurance, the height and weight measurements for a random 5% sample of participants were repeated by a supervisor.

Field-workers visited households every month for 2 years to ensure the interventions were in working condition and being used properly. A short questionnaire was given to caregivers to ascertain adherence to appropriate use of the interventions. Every 3 months, the home visit was extended to measure height and weight and collect a stool sample from the primary study child. A questionnaire was given to caregivers to ascertain water sources and use practices, adherence to the interventions, and for the primary study child: feeding practices, illnesses including diarrhea in the past 7 days, and antibiotic use.

To validate self-reported data on the use of the interventions, at the beginning of the second year of follow-up, we replaced the spigots on the intervention containers with the Smart Spout, a modified spigot with a sensor (an accelerometer, microcontroller, and battery) that measured objective intervention usage based on the duration of each time the spigot was opened. Collection of these data occurred between July 15 and September 15, 2017 by wireless transfer from the spigots to a smart phone.

### Microbiologic methods.

Stool specimens were stored in frozen unpreserved aliquots at −70°C before testing. DNA was extracted from stool using the Qiagen QIAmp Fast DNA Stool Mini Kit (Qiagen, Hilden, Germany) with a modified protocol as previously described^31^ and tested for enteropathogens using multiplex real-time PCR (LightCycler 480, Roche Applied Science, Penzberg, Germany). Targeted genes for amplification identified enteroaggregative *Escherichia coli* (EAEC), enterohemorrhagic *E. coli*/enteropathogenic *E. coli* (EHEC/EPEC), *Giardia*, *Campylobacter jejuni*/*C. coli*, *Cryptosporidium*, enterotoxigenic *E. coli*, *Shigella*/enteroinvasive *E. coli* (EIEC), and adenovirus (Supplemental Table S1).^32^

Microbiological water testing was conducted every 6 months in treated water samples from a random subset of 25 households per intervention group. Membrane filtration was used to enumerate total coliform bacteria and *E. coli* (U.S.E.P.A. method 8074 or 10023). Silver levels in water treated by the silver-impregnated ceramic filters and ceramic tablets were monitored in a random subset of 50–100 households every 3 months.^25^ Total silver concentration was measured by graphite furnace atomic absorption spectrometry (U.S.E.P.A. method 7010). Details of these methods and the results have been reported previously.^25^

### Outcomes.

The primary outcome was the change in ΔHAZ from baseline to the end of follow-up at 24 months of age (HAZ_24 months_ −HAZ_baseline_) in the primary study child. Anthropometric measurements of height and weight were used to construct indices of HAZ, weight-for-height *z*-score (WHZ), and weight-for-age *z*-score (WAZ) using the 2006 WHO child growth standards.^33^ Extreme measurements (identified by the 1st and 99th percentiles of all 3-month changes in *z*-scores) were excluded. If baseline anthropometry was unavailable, then ΔHAZ was calculated as HAZ_24 months_−HAZ_3 months_.

Secondary anthropometric outcomes included ΔWAZ, and ΔWHZ, calculated as above and risk of stunting (HAZ < −2) at 24 months. Total coliform bacteria and *E. coli* in household water samples, 7-day prevalence of diarrhea, and prevalence of enteric infections were also secondary outcomes. We assessed the eight pathogens individually as well as a combined metric of the total count of pathogens detected.

### Data analysis.

The primary analysis was intention-to-treat such that participants were analyzed according to their randomized assignment, regardless of adherence and intervention changes over follow-up. We also conducted a secondary as-treated analysis for the diarrhea and enteric infection outcomes in which participants were analyzed according to the intervention they had at the time of outcome measurement (e.g., participants originally in the ceramic tablet group were included in the filter group after the tablets were replaced with filters).

Baseline household, caregiver, and child characteristics were compared by intervention group and dropout status using descriptive statistics. Adherence to interventions was described based on the self-reported questionnaire data. Households were classified as having objectively used the intervention on a given day if the Smart Spout spigot was held open for at least 5 seconds, which corresponds to approximately 300 mL withdrawn from the container. We included observations from each group on each day in which at least 33 observations were available to reduce the influence of outlier households.

We compared the primary outcome, ΔHAZ, in pairwise comparisons between the four study groups using linear regression, adjusting for age at baseline using cubic splines with 4 knots. We also estimated effects separately by age-group at baseline. We estimated the effects of the interventions on risk of stunting using log-binomial regression adjusting for age as above and a quadratic term for baseline length-for-age *z*-score. We estimated effects on 7-day diarrhea prevalence at all 3-month follow-up visits and on prevalence of enteric infections at 6, 12, 18, and 24 months of follow-up using log-binomial regression adjusting for age and with general estimating equations to account for clustering within repeated measurements from individuals. Estimates for enteric infections were also adjusted for year of stool testing. We conducted a sensitivity analysis in which we excluded samples tested in 2016 and 2017 (15–23% of all follow-up samples depending on the pathogen) based on higher than expected prevalences among these samples.

For all outcomes, based on our prespecified analysis plan, if there was no evidence of effect heterogeneity between the two intervention groups and two control groups, then we assessed the overall impact of the interventions by estimating effects after combining the intervention groups (tablet and filter) and control groups (safe storage container and no intervention).

## RESULTS

A total of 404 households were enrolled and randomized to receive a silver-impregnated ceramic water filter (*n* = 102), a silver-impregnated ceramic tablet (*n* = 99), a safe storage container (*n* = 105), or no intervention (*n* = 98). Almost all mothers (*n* = 390, 96.5%) completed at least secondary school education and were on average 28 years old (SD: 6.7; Table 1). The mean monthly income for the household was 1920 South African Rand (ZAR) (approximately USD135 in June 2016), which is consistent with that of previous studies in the area,^24^ and households had on average 2.4 children younger than 15 years (SD: 1.2). The majority of participants most frequently obtained drinking water from the municipality (piped into their yard, *n* = 91, 22.5%; or from a public stand pipe, *n* = 77, 19.1%) or from surface water through a piped system (into their yard, *n* = 100, 24.8%; or from a public stand pipe, *n* = 46, 11.4%). Municipal water was generated from a treatment facility that uses standard treatment including chlorine disinfection. However, we have previously shown that water from the municipality had little to no detectable residual chlorine by the time it reached the user.^34^ Few households obtained drinking water from groundwater (*n* = 37, 9.2% from springs and *n* = 11, 2.7% from boreholes) or directly from surface water (*n* = 27, 6.7%). Treatment of drinking water was rare (*n* = 62, 15.3%), but most storage vessels were covered (*n* = 328, 81.2%; Table 1). Although most caregivers of children in the youngest age-group (0–11 months) reported that their child was breastfed at the 3-month visits (*n* = 173/205, 84.4%), almost all also reported giving other milks and/or plain water (*n* = 196/205, 95.6%), suggesting exclusive breastfeeding was highly uncommon. Breastfeeding was less common among the older children (*n* = 291/692, 42.1% among ages 12–23 months and *n* = 22/1,650, 1.3% among ages 24+ months).

**Table 1:** Baseline characteristics of 404 enrolled children by intervention group

	Filter (*n* = 102)	Ceramic tablet (*n* = 99)	Safe storage (*n* = 105)	No intervention (*n* = 98)	Overall (*n* = 404)
Demographic/household characteristics					
Mother’s age (years), mean (±SD)	28.4 (±7.1)	27.0 (±6.5)	28.2 (±6.3)	27.8 (±6.8)	27.8 (±6.7)
Highest school grade level of mother, *n* (%)					
Primary	4 (3.9)	5 (5.1)	4 (3.8)	1 (1.0)	14 (3.5)
Secondary	61 (59.8)	55 (55.6)	64 (61.0)	57 (58.2)	237 (58.7)
Matriculation	24 (23.5)	26 (26.3)	21 (20.0)	26 (26.5)	97 (24.0)
Undergraduate	7 (6.9)	10 (10.1)	11 (10.5)	8 (8.2)	36 (8.9)
Postgraduate	4 (3.9)	1 (1.0)	5 (4.8)	5 (5.1)	15 (3.7)
Missing	2 (2.0)	2 (2.0)	0 (0)	1 (1.0)	5 (1.2)
Highest school grade level of the head of the household, *n* (%)					
None	6 (5.9)	4 (4.0)	5 (4.8)	6 (6.1)	21 (5.2)
Primary	24 (23.5)	26 (26.3)	29 (27.6)	24 (24.5)	103 (25.5)
Secondary	47 (46.1)	52 (52.5)	40 (38.1)	40 (40.8)	179 (44.3)
Matriculation	16 (15.7)	11 (11.1)	21 (20.0)	19 (19.4)	67 (16.6)
Undergraduate	6 (5.9)	2 (2.0)	6 (5.7)	4 (4.1)	18 (4.5)
Postgraduate	3 (2.9)	4 (4.0)	4 (3.8)	5 (5.1)	16 (4.0)
Relationship of the head of the household to the child, *n* (%)					
Father	37 (36.3)	33 (33.3)	38 (36.2)	27 (27.6)	135 (33.4)
Mother	15 (14.7)	13 (13.1)	13 (12.4)	21 (21.4)	62 (15.3)
Grandmother	32 (31.4)	29 (29.3)	32 (30.5)	31 (31.6)	124 (30.7)
Grandfather	15 (14.7)	24 (24.2)	20 (19.0)	15 (15.3)	74 (18.3)
Sibling	0 (0.0)	0 (0.0)	0 (0.0)	1 (1.0)	1 (0.2)
Other	3 (2.9)	0 (0.0)	2 (1.9)	3 (3.1)	8 (2.0)
Socioeconomic status score [WAMI; mean (±SD)]	0.79 (±0.11)	0.78 (±0.12)	0.78 (±0.13)	0.78 (±0.11)	0.78 (±0.11)
Monthly household income (ZAR), mean (±SD)	1,746 (±1,154)	1,987 (±1,865)	2,300 (±3,066)	1,626 (±886)	1,920 (±1,965)
Adults older than 15 years in household, mean (±SD)	2.9 (±1.2)	3.1 (±1.6)	3.1 (±1.4)	2.9 (±1.6)	3.0 (±1.5)
Children younger than 15 years in household, mean (±SD)	2.3 (±1.0)	2.3 (±1.1)	2.5 (±1.4)	2.5 (±1.2)	2.4 (±1.2)
Crowded household (> 2/room for sleeping), *n* (%)	47 (46.1)	47 (47.5)	54 (51.4)	55 (56.1)	203 (50.2)
Water use practices					
Primary drinking water source, *n* (%)					
Municipal	38 (37.3)	47 (47.5)	39 (37.1)	44 (44.9)	168 (41.6)
Surface water from tap/pipe	41 (40.2)	32 (32.3)	41 (39.0)	32 (32.7)	146 (36.1)
Directly from surface water	7 (6.9)	5 (5.1)	9 (8.6)	6 (6.1)	27 (6.7)
Groundwater	11 (10.8)	15 (15.2)	10 (9.5)	12 (12.2)	48 (11.9)
Unknown/other	5 (4.9)	0 (0.0)	6 (5.7)	4 (4.1)	15 (3.7)
Length of time to collect water (minutes), mean (±SD)	24.6 (±43.2)	31.2 (±57.9)	26.9 (±43.3)	24.4 (±34.8)	26.7 (±45.4)
Typical point-of-use drinking water treatment, *n* (%)					
Let stand and settle	2 (2.0)	3 (3.0)	2 (1.9)	4 (4.1)	11 (2.7)
Add bleach/chlorine	2 (2.0)	6 (6.1)	2 (1.9)	5 (5.1)	15 (3.7)
Boil	6 (5.9)	5 (5.1)	14 (13.3)	8 (8.2)	33 (8.2)
Other	1 (1.0)	1 (1.0)	0 (0.0)	1 (1.0)	3 (0.7)
None	91 (89.2)	84 (84.8)	87 (82.9)	80 (81.6)	342 (84.7)
Covered water storage vessels, *n* (%)	82 (80.4)	84 (84.8)	86 (81.9)	76 (77.6)	328 (81.2)
Main water supply, *n* (%)					
Continuous	30 (29.4)	19 (19.2)	33 (31.4)	21 (21.4)	103 (25.5)
Sometimes interrupted	72 (70.6)	80 (80.8)	72 (68.6)	77 (78.6)	301 (74.5)
Improved toilet facility, *n* (%)					
Unimproved	8 (7.8)	6 (6.1)	8 (7.6)	3 (3.1)	25 (6.2)
Improved	94 (92.2)	93 (93.9)	97 (92.4)	95 (96.9)	379 (93.8)
Frequency of handwashing after using toilet, *n* (%)					
Never	5 (4.9)	2 (2.0)	2 (1.9)	5 (5.1)	14 (3.5)
Rarely	12 (11.8)	16 (16.2)	11 (10.5)	17 (17.3)	56 (13.9)
Often	7 (6.9)	13 (13.1)	10 (9.5)	8 (8.2)	38 (9.4)
Always	78 (76.5)	68 (68.7)	82 (78.1)	68 (69.4)	296 (73.3)
Child characteristics, *n* (%)					
Diarrhea in primary study child in the last 7 days	20 (19.6)	23 (23.2)	22 (21.0)	20 (20.4)	85 (21.0)
Age of primary study child at baseline (years), *n* (%)					
< 1	42 (41.2)	38 (38.4)	40 (38.1)	35 (35.7)	155 (38.4)
1–2	37 (36.3)	34 (34.3)	41 (39.0)	36 (36.7)	148 (36.6)
2–3	23 (22.5)	27 (27.3)	24 (22.9)	27 (27.6)	101 (25.0)
Length/height-for-age *z*-score at baseline, mean (±SD)*	−1.25 (±1.23)	−1.19 (±1.38)	−1.44 (±1.12)	−1.63 (±1.35)	−1.38 (±1.28)
Weight-for-age *z*-score at baseline, mean (±SD)*	−0.21 (±1.11)	−0.25 (±1.19)	−0.42 (±1.19)	−0.48 (±1.38)	−0.34 (±1.22)
Height-for-weight *z*-score at baseline, mean (±SD)*	0.69 (±1.25)	0.54 (±1.40)	0.55 (±1.48)	0.49 (±1.50)	0.56 (±1.41)
Stunted at baseline, *n* (%)*	25 (24.5)	29 (29.3)	31 (29.5)	40 (40.8)	125 (30.9)
Underweight at baseline, *n* (%)*	7 (6.9)	7 (7.1)	9 (8.6)	11 (11.2)	34 (8.4)
Wasted at baseline, *n* (%)*	2 (2.0)	5 (5.1)	4 (3.8)	4 (4.1)	15 (3.7)

* Baseline length/height unavailable for 10 (2.5%) children; baseline weight unavailable for 1 (0.2%) child.

**Figure 1.**
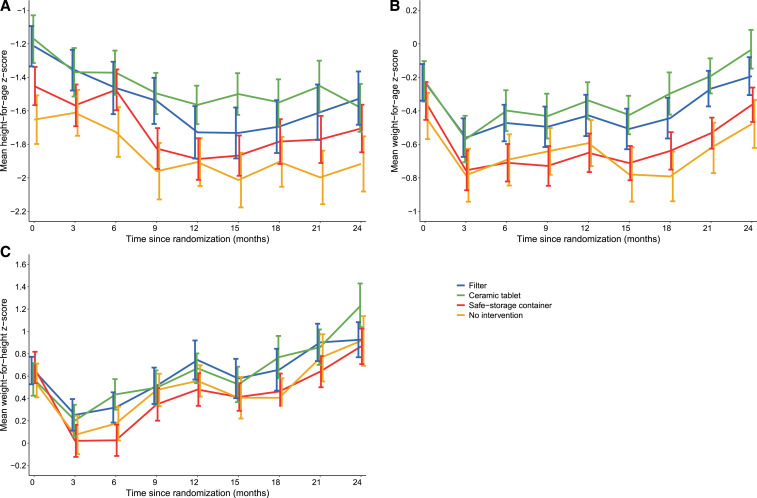
Mean height-for-age (**A**), weight-for-age (**B**), and weight-for-height (**C**) *z*-score growth trajectories among 404 children. Error bars represent standard errors. This figure appears in color at www.ajtmh.org.

Baseline household characteristics were similar between intervention arms. However, children in the no intervention group were slightly older, and mean anthropometric *z*-scores were lower (Table 1). A total of 114 (28.2%) household dropped out (*n* = 57, 14.1% in each year), such that 290 households completed the 24-month follow-up visit (Figure 1). Dropout was not significantly associated with randomization group (*P* = 0.6), but children who dropped out were younger, their anthropometric *z*-scores were lower at baseline, and their mothers were slightly less educated than children who completed follow-up (Supplemental Table S2). For example, 39% (*n* = 44) of children who dropped out were stunted at baseline compared with 28% (*n* = 81) of children who completed follow-up.

### Adherence.

Participants randomized to one of the three intervention groups self-reported adherence data a median of 19 times (interquartile range [IQR]: 16, 21) at monthly visits over 2 years of follow-up. At most of these visits, the participants reported that members of the household drank water from the intervention container 7 days per week (88.3%, *n* = 4,552/5,121), and this proportion was higher among participants randomized to receive the ceramic tablet (92.7%) than among participants randomized to receive the filter (85.6%) or the safe storage container (86.7%; *P* < 0.0001). Participants reported that someone in the household drank water from the intervention container specifically during the previous day at 96.7% of visits (*n* = 4,952/5,122), and this proportion did not differ by the intervention group (*P* = 0.2). The proportion drinking from the intervention container on the previous day was 90% or higher every month and increased on average by 2.6% (95% CI: 0.5, 4.7) from baseline to 24 months. Participants reported refilling the intervention container a median of every 3 days (IQR: 3, 4), almost exclusively by an adult woman (96.7%, *n* = 1,888/1,953) in the morning (62.6%, *n* = 1,223/1,953) or at night (23.1%, *n* = 452/1,953).

The Smart Spout measured less intervention usage (Supplemental Figure S1). On an average day, 75.8% of participants used their interventions, including 71.5% in the ceramic filter group, 81.2% in the ceramic tablet group, and 81.4% in the safe storage container alone group. After households in the ceramic tablet group were switched to the filter intervention, use on an average day in this group dropped to 68.1%. Consistent intervention use was less common; although on an average day 80.1% of households had used the intervention on at least 4 of the past 7 days, only 43.4% of households had used their intervention on each of the previous 7 days (39.1%, 44.2%, and 51.4% in the filter, tablet, and safe storage container groups, respectively; Supplemental Figure S1).

Caregivers reported that the primary study child most often drank water from the intervention container at 97.5% of visits. The primary study child additionally drank water from other sources or storage containers while at home at 18.2% (*n* = 934/5,122) of monthly visits, primarily because caregivers forgot about the intervention (*n* = 858, 91.9%), and rarely because the container was empty (*n* = 23, 2.5%), the intervention was broken (*n* = 12, 1.3%), or they disliked the taste or smell of the water (*n* = 7, 0.8%). The proportion of children drinking water from other sources did not change over the study period (*P* = 0.5), but was lower in the ceramic tablet group (9.8%) than the filter (22.9%) and safe storage container (21.8%) groups (*P* < 0.0001).

### Water quality.

Intervention effects on water quality were previously reported.^25–29^ We evaluated the effect of the interventions in 150 drinking water samples taken at 0, 6, and 12 months from households that had a ceramic filter (*n* = 75) or tablet (*n* = 75). In summary, the mean total coliform concentrations in household drinking water before treatment by the silver-impregnated ceramic filter and tablet were 4,654 cfu/100 mL and 5,722 cfu/100 mL, respectively. The filters and tablets provided approximately 1.2 and 3 log reductions of total coliform bacteria in the treated drinking water, respectively.^25^ Only seven (9.3%) water samples treated by the tablet had 1 cfu/100 mL or greater, and 30 (40.0%) water samples treated by the silver-impregnated ceramic filter had 1 cfu/100 mL or greater.^25^

### Anthropometry.

At baseline, children in the first year of life had a mean HAZ of −0.79 (SD: 1.31) and WAZ of −0.01 (SD: 1.29). Children in the second and third years of life were further below the WHO growth standards with mean HAZ of −1.66 (SD: 1.11) and −1.77 (SD: 1.20), and WAZ of −0.47 (SD: 1.19) and −0.49 (SD: 0.94), respectively. Adjusting for age, children in the filter group were 0.42 *z*-scores (95% CI: 0.08, 0.76) taller at baseline than children in the no intervention group (Supplemental Table S3). Similarly, children in the ceramic tablet (mean difference (MD): 0.46, 95% CI: 0.12, 0.80) and safe storage container (MD: 0.21, 95% CI: −0.13, 0.55) groups were taller than those in the no intervention group at baseline. WAZ were also higher in the intervention groups, but these differences were not statistically significant (Supplemental Table S3).

Mean growth trajectories of children in the four intervention groups are shown in Figure 1. Children in the filter, ceramic tablet, and safe storage container groups were taller than those in the no intervention group at all time points. Similar differences were observed for WAZ and WHZ. The mean change over the two years of follow-up was −0.28 (SD = 1.24) for ΔHAZ, 0.05 (SD = 0.99) for ΔWAZ, and 0.34 (SD = 1.56) for ΔWHZ. Adjusting for age, there were no differences in ΔHAZ, ΔWAZ, or ΔWHZ among children in the intervention groups compared with children in the no intervention group (Table 2). Similarly, there were no differences between the combined intervention (filter and ceramic tablet) and combined control (safe storage container and no intervention groups). Finally, there were no differences in ΔHAZ, ΔWAZ, or ΔWHZ when stratifying by age at baseline (Supplemental Table S4).

**Table 2:** Effect of water treatment interventions on child growth among 288 children who completed 24 months of follow-up and had a baseline anthropometric measure

Intervention	Number of children	*Z*-score at baseline,mean (SD)	*Z*-score at 24 months, mean (SD)	Δ*Z*-score, mean (SD)	Mean Δ*Z*-score difference,* (95% CI)
Height-for-age *z*-score					
Filter	70	−1.08 (1.15)	−1.42 (1.05)	−0.35 (1.20)	0.06 (−0.29, 0.40)
Ceramic tablet	69	−1.24 (1.31)	−1.53 (1.12)	−0.29 (1.31)	0.00 (−0.35, 0.35)
Safe storage	75	−1.44 (1.13)	−1.63 (1.13)	−0.19 (1.16)	0.11 (−0.23, 0.44)
No intervention	62	−1.60 (1.39)	−1.88 (1.29)	−0.28 (1.30)	0.
Combined intervention	139	−1.16 (1.23)	−1.48 (1.08)	−0.32 (1.25)	−0.03 (−0.27, 0.21)
Combined control	137	−1.51 (1.25)	−1.75 (1.20)	−0.23 (1.22)	0.
Weight-for-age *z*-score					
Filter	70	−0.13 (1.06)	−0.19 (0.95)	−0.06 (0.97)	−0.08 (−0.42, 0.26)
Ceramic tablet	70	−0.22 (1.21)	−0.05 (0.97)	0.16 (1.03)	0.13 (−0.21, 0.46)
Safe storage	79	−0.40 (1.14)	−0.36 (0.91)	0.04 (0.94)	−0.04 (−0.36, 0.29)
No intervention	63	−0.52 (1.26)	−0.48 (1.14)	0.04 (1.03)	0.
Combined intervention	140	−0.17 (1.13)	−0.12 (0.95)	0.05 (1.01)	0.04 (−0.19, 0.27)
Combined control	142	−0.45 (1.19)	−0.41 (1.02)	0.04 (0.97)	0.
Weight-for-height *z*-score					
Filter	68	0.64 (1.23)	0.93 (1.29)	0.29 (1.33)	−0.11 (−0.65, 0.43)
Ceramic tablet	68	0.63 (1.44)	1.17 (1.60)	0.54 (1.75)	0.13 (−0.41, 0.67)
Safe storage	74	0.62 (1.33)	0.80 (1.34)	0.18 (1.44)	−0.24 (−0.77, 0.29)
No intervention	60	0.49 (1.49)	0.86 (1.70)	0.38 (1.72)	0.
Combined intervention	136	0.63 (1.34)	1.05 (1.46)	0.41 (1.55)	0.14 (−0.23, 0.51)
Combined control	134	0.56 (1.40)	0.83 (1.51)	0.27 (1.57)	0.

* Adjusted for age using cubic splines with 4 knots.

More than a quarter (*n* = 82, 29.7%) of children were stunted after 2 years of follow-up. Children in the filter, ceramic tablet, and safe storage container groups had a lower risk of stunting after 2 years than those in the no intervention group, but these estimates were not statistically significant and likely reflect the large differences in prevalence in stunting at baseline, despite adjustment for age and HAZ at baseline (Supplemental Table S5). Only 11 and four children were underweight and wasted at 24 months, respectively, such that comparison across intervention groups for these secondary outcomes was not possible.

### Diarrhea prevalence.

**Figure 2.**
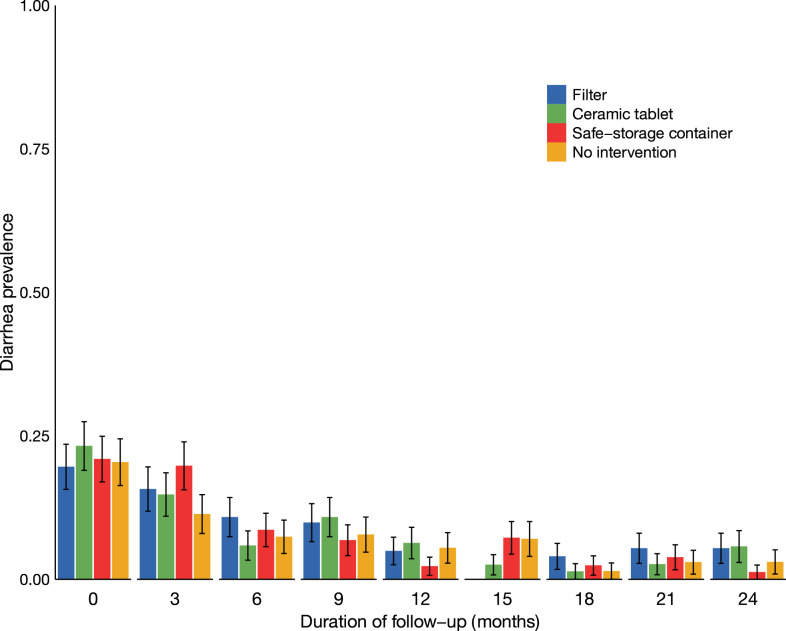
Seven-day prevalence of diarrhea by intervention group and duration of follow-up among 404 children. Error bars represent standard errors. This figure appears in color at www.ajtmh.org.

The 7-day prevalence of diarrhea at baseline was 18.5% (*n* = 23), 22.7% (*n* = 23), and 21.6% (*n* = 30) among children in their first, second, and third years of life, respectively. There were no significant differences in diarrhea prevalence at baseline between intervention groups (*P* = 0.9). Diarrhea prevalence decreased over the 2 years of follow-up in all intervention groups (Figure 2). At 2 years of follow-up, 3.8% (*n* = 11) of children reported diarrhea in the past 7 days. There were no significant differences in diarrhea prevalence between intervention groups Table 3. The prevalence of diarrhea in the combined intervention group was 1.05 times (95% CI: 0.73, 1.50) the prevalence in the combined control group. In a secondary as-treated analysis, there were no differences in diarrhea prevalence between intervention groups (Table 2).

**Table 3:** Intervention effects on 7-day prevalence of diarrhea at quarterly follow-up visits for 24 months among 388 children

Intervention	Intention-to-treat analysis			As-treated analysis		
	Number of follow-up visits	Diarrhea in the past 7 days, *n* (%)	Prevalence ratio* (95% CI)	Number of follow-up visits	Diarrhea in the past 7 days, *n* (%)	Prevalence ratio* (95% CI)
Filter	643	46 (7.2)	1.17 (0.70, 1.96)	490	39 (8.1)	1.14 (0.67, 1.94)
No silver filter	–	–	–	467	18 (3.9)	1.09 (0.52, 2.30)
Ceramic tablet	634	41 (6.5)	1.07 (0.61, 1.88)	320	30 (9.4)	1.23 (0.69, 2.21)
Safe storage	688	46 (6.7)	1.13 (0.69, 1.84)	688	46 (6.7)	1.13 (0.69, 1.84)
No intervention	598	36 (6.1)	1.	598	36 (6.1)	1.
Combined intervention†	1,277	87 (6.9)	1.05 (0.73, 1.50)	810	69 (8.5)	1.10 (0.75, 1.61)
Combined control	1,286	82 (6.4)	1.	1,286	82 (6.4)	1.

* Adjusted for age using cubic splines with 4 knots.

† For as-treated analysis, excludes the no silver filter group.

### Enteric infections.

For the analysis of enteric infections, we collected 2,654 stool samples quarterly from baseline to 2 years of follow-up and tested 1,670 samples from 0, 6, 12, 18, and 24 months. Valid qPCR results were available for at least one of the pathogens tested in 1,624 (97.2%) stool samples. Children had on average 4.0 (SD: 1.42) stool samples tested. Diarrhea was reported in the previous 7 days for 139 (8.6%) samples, and there was no difference in the proportion of stools collected within 7 days of diarrhea across intervention arms (*P* = 0.7).

**Figure 3.**
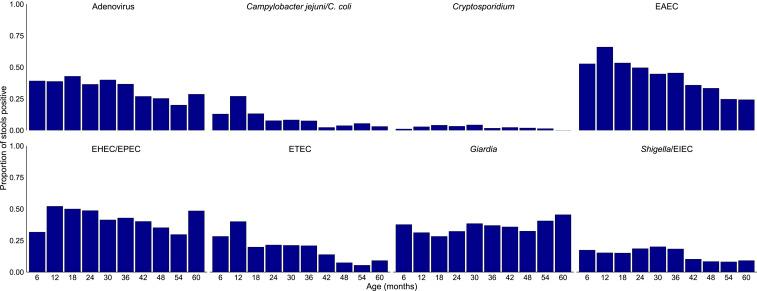
Prevalence of enteric pathogens by age among 394 children with at least one stool validly tested. *E. coli* = *Escherichia coli*; EAEC = enteroaggregative *E. coli*, EHEC = enterohemorrhagic *E. coli*, EIEC = enteroinvasive *E. coli*, EPEC: enteropathogenic *E. coli*, ETEC = enterotoxigenic *E. coli*. This figure appears in color at www.ajtmh.org.

Enteric infections were highly common (Figure 3). Most stool samples (*n* = 1,376, 86.3%) were positive for at least one of the eight enteric pathogens detected, and the mean number of pathogens detected per sample was 2.04 (SD: 1.53). Enteroaggregative *Escherichia coli* (*n* = 746, 46.0%), EHEC/EPEC (*n* = 658, 43.2%), and adenovirus (*n* = 549, 35.8%) were the most commonly detected pathogens. There were no significant differences in enteric infection prevalence at baseline (Supplemental Figure S2).

**Figure 4.**
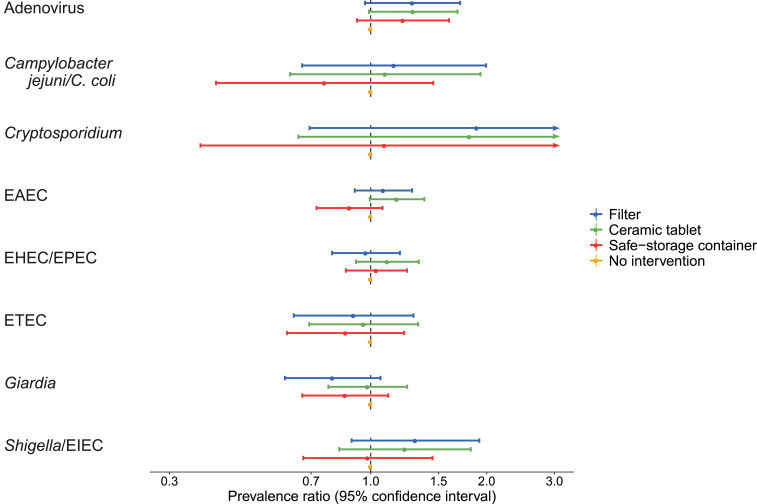
Effects of the silver-impregnated ceramic filter, silver-impregnated ceramic tablet, and safe storage water container on the prevalence of enteric infections compared with no intervention among 365 children with at least one stool validly tested after the baseline visit. *E. coli* = *Escherichia coli*; EAEC = enteroaggregative *E. coli*, EHEC = enterohemorrhagic *E. coli*, EIEC = enteroinvasive *E. coli*, EPEC = enteropathogenic *E. coli*; ETEC = enterotoxigenic *E. coli*. This figure appears in color at www.ajtmh.org.

The interventions had no statistically significant effects on the prevalence of any of the eight enteric pathogens between 6 and 24 months of follow-up (Figure 4). Adenovirus, *Cryptosporidium*, and *Shigella*/EIEC were more common in the intervention groups than in the no intervention group, but estimates were imprecise. Conversely, *Giardia* was less common in the filter group than in the no intervention group, but this difference was also not significant. There were no differences in the total number of pathogens detected between individual intervention groups (Supplemental Table S6). When combining intervention and control groups, there was a 20% higher prevalence of EAEC (PR: 1.20, 95% CI: 1.06, 1.36) in the intervention groups than in the control (Supplemental Table S6). The total number of pathogens detected was also 11% higher in the combined intervention group than in the combined control group (ratio of number of pathogens detected: 1.11, 95% CI: 1.02, 1.22).

In a secondary as-treated analysis, there were no differences in enteric pathogen prevalence between intervention groups, except that there was a higher frequency of EAEC in the combined intervention than in the combined control groups (Supplemental Table S7). Results were consistent in a sensitivity analysis in which samples tested in 2016 and 2017 were excluded, except that there was also a higher frequency of *Cryptosporidium* in the combined intervention than in the combined control groups (Supplemental Table S8).

## DISCUSSION

Although the ceramic filters and MadiDrops significantly reduced the total coliform bacteria in treated water, the improvements in household drinking water quality did not translate to significant improvements in child growth. No differences were observed in ΔHAZ, ΔWAZ, or ΔWHZ among children in the intervention groups compared with children in the no intervention group. Similarly, access to the silver-impregnated ceramic filter or tablet did not prevent enteric infections. Despite the high frequency of infections in this population, there were no statistically significant effects of the interventions on the prevalence of any of the eight enteric pathogens or the total number of pathogens detected between 6 and 24 months of follow-up. The expected mechanism for the impact of drinking water interventions on linear growth is through a reduction in enteric infections. The lack of effect on linear growth may therefore be explained by no significant reductions in enteric infections.

There was also no effect on diarrhea prevalence in both intention-to-treat and as-treated analyses. The cohort had low diarrhea rates at baseline that declined throughout the study, likely because of the children aging. Therefore, there may have been limited opportunity to interrupt transmission of diarrhea-associated pathogens.

The limited impact on both primary and secondary outcomes observed in this study could be attributed to several explanations. Children are exposed to enteric pathogens through a variety of transmission pathways including contaminated food and exposure to soil contaminated with animal feces.^35–37^ Specifically, enteroaggregative *E. coli* and EPEC can be transmitted through contaminated food in addition to water, and adenovirus and *Shigella*/EIEC are primarily transmitted person-to-person.^38–41^ Preventing transmission only through pathways involving drinking water allows for children to still be exposed through these alternate pathways. In addition, the adherence data showed that all participants did not use their water intervention every day, suggesting that some children could have been exposed to pathogens from untreated drinking water sources. Furthermore, the quantity of water available may have been as or more important than quality because quantity determines how much is used for hygiene, and the volume of water used decreases substantially once water access is off-plot,^42^ which was common in this study. On the other hand, breastfeeding may have provided protection against enteric infections and reduced drinking water requirements for the youngest children across intervention groups. However, exclusive breastfeeding was rare, and breastfeeding did not eliminate exposure to potentially contaminated water in this setting. Finally, the interventions were implemented at the individual level rather than at the community level, which may be required to sufficiently limit pathogen contamination.^43,44^

The focus on objective outcomes, including anthropometric measures and enteric infections, was a strength of the study. Although participants were not masked to the intervention received, all laboratory analyses were blinded to the intervention group. The secondary outcome of caregiver-reported diarrheal prevalence was potentially biased because the caregivers were not blinded to which intervention group they belonged to, which may have resulted in intervention households less likely to report diarrhea than control households.^45^ Objective detection of enteric pathogens helped to avoid recall bias and directly measured part of the causal pathway between the water treatment intervention and growth.^46^ Similarly, the objective adherence data measured through the Smart Spout improved our ability to characterize intervention usage, and, perhaps not surprisingly, objective measures of adherence were lower than the near-universal self-reported adherence.

This study was limited by the small study size and low diarrhea rates, which restricted our ability to detect significant effects of the interventions. Furthermore, the age range of enrolled children was relatively wide, which may have obscured effects in the youngest age-group that has the highest growth rates. Although age-specific effects were highly underpowered, the intervention effects were largest in this subgroup. The inconsistent adherence as measured by the Smart Spout was also a limitation, as high adherence to water quality interventions has been shown to be required for interventions to be effective in achieving gains in health outcomes.^47^ The changes of interventions over follow-up were limiting in that each group did not have the same device throughout the study, and the filters without silver were substantially less effective in treating drinking water than silver-impregnated ceramic tablets and filters.^25^ However, silver-impregnated ceramic tablets with 100% and 50% silver had similar rates of disinfection, suggesting that they should have had similar effectiveness. Because the primary analysis was intention-to-treat, bias from intervention changes would be expected to be toward the null. Difference in height at baseline between intervention groups was also a limitation, but because the primary outcome was change in height, these baseline differences were accounted for in the analysis.

The results on child growth from this trial are consistent with those from several recent large intervention trials (WASH Benefits and SHINE), which were larger and tested more comprehensive WASH and nutrition interventions.^8–10^ Similar to this study, WASH Benefits Bangladesh, WASH Benefits Kenya, and SHINE showed that WASH interventions did not improve linear growth.^8–10^ The interventions in SHINE also did not reduce enteric infections.^48^ Furthermore, WASH Benefits Kenya^9^ and SHINE^10^ found no impact on diarrhea, and diarrheal prevalence was reduced in all intervention arms of the WASH Benefits Bangladesh trial, except in the drinking water treatment alone arm.^8^ Importantly, these studies all tested POU water chlorination, which is a much more labor-intensive intervention than the ceramic filter and tablet tested here. The Smart Spout documented that adherence was higher for the ceramic tablet than for the filter, potentially because the tablet requires less user interaction. Demands on the user are an important design consideration affecting uptake. This study demonstrates that POU interventions that were less demanding of the user were still ineffective in reducing enteropathogen transmission. Therefore, our results support the consensus conclusion from the previous studies that more transformative interventions, including larger scale infrastructure projects, are likely necessary to prevent enteric infections and improve growth among children.^43^

Although this study and the WASH Benefits and SHINE trials did not show effectiveness to prevent enteric infections or growth stunting, several studies have shown that POU interventions reduce the incidence of diarrheal disease in diverse settings.^16–18,49^ Specifically, fabric filtration yielded a 48% reduction in cholera in a study in Bangladesh with 133,000 participants. In addition, silver-impregnated ceramic water filters were shown to reduce diarrheal prevalence in HIV-positive individuals in Limpopo, South Africa.^15^ However, these studies involved special populations that may have had higher adherence than families of healthy young children. Regardless, improving access to safe drinking water is important not only for the health impacts but also because it promotes dignity and protects the human right of access to safe drinking water.^50^

Despite their effectiveness in treating drinking water, the use of the silver-impregnated ceramic water filters and tablets did not reduce enteric infections or improve child growth in this trial. More comprehensive WASH interventions, potentially at the community level, that better prevent enteric infections are likely needed to demonstrate improvements in long-term child growth outcomes.

## Supplemental materials

Supplemental materials
