# Supplementary material for: Impact of Low-Cost Point-of-Use Water Treatment Technologies on Enteric Infections and Growth among Children in Limpopo, South Africa
Source: Am J Trop Med Hyg. 2020 Aug 24;103(4):1405–15. doi: 10.4269/ajtmh.20-0228 (PMC7543807; doi:10.4269/ajtmh.20-0228)
Supplement: Supplementary file 1 [file tpmd200228.SD1.pdf]

Supplemental material

Impact of low-cost point-of-use water treatment technologies on enteric infections and linear growth among children in Limpopo, South Africa

Courtney L. Hill, Kelly McCain, Mzwakhe E. Nyathi, Joshua N. Edokpayi, David M. Kahler, Darwin J. Operario, David D.J. Taylor, Natasha C. Wright, James A. Smith, Richard L. Guerrant, Amidou Samie, Rebecca A. Dillingham, Pascal O. Bessong, and Elizabeth T. Rogawski McQuade

Table S1. Targeted genes for amplification in real-time PCR assays.

	Pathogen	Gene
Virus	Adenovirus	Hexon gene
Bacteria	EAEC*	*aaiC, aatA*
	EHEC/EPEC*	*eae*
	ETEC*	*LT*, *STh* or *STp*
	Campylobacter jejuni/ C. coli	*cadF*
	Shigella/EIEC	*ipaH*
Protozoa	*Cryptosporidium*	18S rRNA
	Giardia	18S rRNA

**E. coli* pathotypes were defined as follows: EAEC (*aaiC*, or *aatA*, or both), EHEC/EPEC (*eae*; includes typical and atypical EPEC), ETEC (STh, STp, or LT).

EAEC: enteroaggregative E. coli; EHEC: enterohemorrhagic E. coli; EPEC: enteropathogenic E. coli; ETEC: enterotoxigenic E. coli; EIEC: enteroinvasive E. coli

Table S2. Baseline characteristics of 404 study participants who did and did not complete follow-up to 24 months.

	Completed follow-up (n=290)	Dropped out before 24 months (n=114)	Overall (n=404)
Demographic/Household Characteristics			
Mother's age (years)			
Mean (SD)	28.3 (± 6.8)	26.7 (± 6.2)	27.8 (± 6.7)
Highest school grade level of mother, n (%)			
Primary	10 (3.4%)	4 (3.5%)	14 (3.5%)
Secondary	166 (57.2%)	71 (62.3%)	237 (58.7%)
Matriculation	71 (24.5%)	26 (22.8%)	97 (24.0%)
Undergraduate	27 (9.3%)	9 (7.9%)	36 (8.9%)
Post-graduate	13 (4.5%)	2 (1.8%)	15 (3.7%)
Missing	3 (1.0%)	2 (1.8%)	5 (1.2%)
Highest school grade level of head of household, n (%)			
None	16 (5.5%)	5 (4.4%)	21 (5.2%)
Primary	68 (23.4%)	35 (30.7%)	103 (25.5%)
Secondary	131 (45.2%)	48 (42.1%)	179 (44.3%)
Matriculation	51 (17.6%)	16 (14.0%)	67 (16.6%)
Undergraduate	13 (4.5%)	5 (4.4%)	18 (4.5%)
Post-graduate	11 (3.8%)	5 (4.4%)	16 (4.0%)
Relationship of head of household to child, n (%)			
Father	104 (35.9%)	31 (27.2%)	135 (33.4%)
Mother	38 (13.1%)	24 (21.1%)	62 (15.3%)
Grandmother	88 (30.3%)	36 (31.6%)	124 (30.7%)
Grandfather	56 (19.3%)	18 (15.8%)	74 (18.3%)
Sibling	0 (0.0%)	1 (0.9%)	1 (0.2%)
Other	4 (1.4%)	4 (3.5%)	8 (2.0%)
Socioeconomic Status Score (WAMI)			
Mean (SD)	0.79 (± 0.11)	0.78 (± 0.12)	0.78 (± 0.11)
Monthly household income (ZAR)			
Mean (SD)	1932 (± 2047)	1889 (± 1748)	1920 (± 1965)
Adults over 15 in household			
Mean (SD)	3.1 (± 1.5)	2.9 (± 1.4)	3.0 (± 1.5)
Children under 15 in household			
Mean (SD)	2.4 (± 1.2)	2.4 (± 1.2)	2.4 (± 1.2)
Access to electricity in household, n (%)			
No	10 (3.4%)	6 (5.3%)	16 (4.0%)
Yes	280 (96.6%)	108 (94.7%)	388 (96.0%)
Crowded household (>2/room for sleeping), n (%)			
No	146 (50.3%)	55 (48.2%)	201 (49.8%)

Yes	144 (49.7%)	59 (51.8%)	203 (50.2%)
Main material of house floor, n (%)			
Earth/sand/clay/mud/dung	6 (2.1%)	3 (2.6%)	9 (2.2%)
Ceramic tiles or vinyl	23 (7.9%)	6 (5.3%)	29 (7.2%)
Cement or concrete	261 (90.0%)	105 (92.1%)	366 (90.6%)
Water Use Practices			
Primary drinking water source, n (%)			
Municipal	111 (38.3%)	57 (50.0%)	168 (41.6%)
Surface water from tap/pipe	110 (37.9%)	36 (31.6%)	146 (36.1%)
Directly from surface water	18 (6.2%)	9 (7.9%)	27 (6.7%)
Groundwater	43 (14.8%)	5 (4.4%)	48 (11.9%)
Unknown/Other	8 (2.8%)	7 (6.1%)	15 (3.7%)
Length of time to collect water (minutes)			
Mean (SD)	29.7 (± 49.9)	19.2 (± 30.5)	26.7 (± 45.4)
Typical point-of-use drinking water treatment, n (%)			
Let stand and settle	6 (2.1%)	5 (4.4%)	11 (2.7%)
Add bleach/chlorine	11 (3.8%)	4 (3.5%)	15 (3.7%)
Boil	24 (8.3%)	9 (7.9%)	33 (8.2%)
Other	2 (0.7%)	1 (0.9%)	3 (0.7%)
None	247 (85.2%)	95 (83.3%)	342 (84.7%)
Drinking water storage vessel type, n (%)			
Metal buckets	6 (2.1%)	5 (4.4%)	11 (2.7%)
Plastic buckets	119 (41.0%)	43 (37.7%)	162 (40.1%)
Jerrycan	145 (50.0%)	55 (48.2%)	200 (49.5%)
Plastic bottles	4 (1.4%)	1 (0.9%)	5 (1.2%)
Other	16 (5.5%)	10 (8.8%)	26 (6.4%)
Covered water storage vessels, n (%)			
No	52 (17.9%)	24 (21.1%)	76 (18.8%)
Yes	238 (82.1%)	90 (78.9%)	328 (81.2%)
Method of collecting drinking water from storage, n (%)			
Pour directly	14 (4.8%)	7 (6.1%)	21 (5.2%)
Use cup with handle	205 (70.7%)	76 (66.7%)	281 (69.6%)
Use cup with hands	63 (21.7%)	29 (25.4%)	92 (22.8%)
Use spigot	3 (1.0%)	1 (0.9%)	4 (1.0%)
Other	5 (1.7%)	1 (0.9%)	6 (1.5%)
Continuous main water supply, n (%)			
Continuous	74 (25.5%)	29 (25.4%)	103 (25.5%)
Sometimes interrupted	216 (74.5%)	85 (74.6%)	301 (74.5%)
Improved toilet facility, n (%)			
Unimproved	19 (6.6%)	6 (5.3%)	25 (6.2%)
Improved	271 (93.4%)	108 (94.7%)	379 (93.8%)

Frequency of handwashing after using toilet, n (%)			
Never	11 (3.8%)	3 (2.6%)	14 (3.5%)
Rarely	41 (14.1%)	15 (13.2%)	56 (13.9%)
Often	31 (10.7%)	7 (6.1%)	38 (9.4%)
Always	207 (71.4%)	89 (78.1%)	296 (73.3%)
Child Characteristics			
Diarrhea in primary study child in last 7 days, n (%)			
No	230 (79.3%)	89 (78.1%)	319 (79.0%)
Yes	60 (20.7%)	25 (21.9%)	85 (21.0%)
Age of primary study child at baseline (years), n (%)			
<1	103 (35.5%)	52 (45.6%)	155 (38.4%)
1-2	107 (36.9%)	41 (36.0%)	148 (36.6%)
2-3	80 (27.6%)	21 (18.4%)	101 (25.0%)
Length/height-for-age z-score at baseline			
Mean (SD)	-1.34 (± 1.26)	-1.46 (± 1.34)	-1.38 (± 1.28)
Missing	9 (3.1%)	1 (0.9%)	10 (2.5%)
Weight-for-age z-score at baseline			
Mean (SD)	-0.32 (± 1.17)	-0.38 (± 1.34)	-0.34 (± 1.22)
Missing	1 (0.3%)	0 (0%)	1 (0.2%)
Height-for-weight z-score at baseline			
Mean (SD)	0.58 (± 1.37)	0.52 (± 1.49)	0.56 (± 1.41)
Missing	10 (3.4%)	1 (0.9%)	11 (2.7%)
Stunted at baseline, n (%)			
No	200 (69.0%)	69 (60.5%)	269 (66.6%)
Yes	81 (27.9%)	44 (38.6%)	125 (30.9%)
Missing	9 (3.1%)	1 (0.9%)	10 (2.5%)
Underweight at baseline, n (%)			
No	265 (91.4%)	104 (91.2%)	369 (91.3%)
Yes	24 (8.3%)	10 (8.8%)	34 (8.4%)
Missing	1 (0.3%)	0 (0%)	1 (0.2%)
Wasted at baseline, n (%)			
No	269 (92.8%)	109 (95.6%)	378 (93.6%)
Yes	11 (3.8%)	4 (3.5%)	15 (3.7%)
Missing	10 (3.4%)	1 (0.9%)	11 (2.7%)

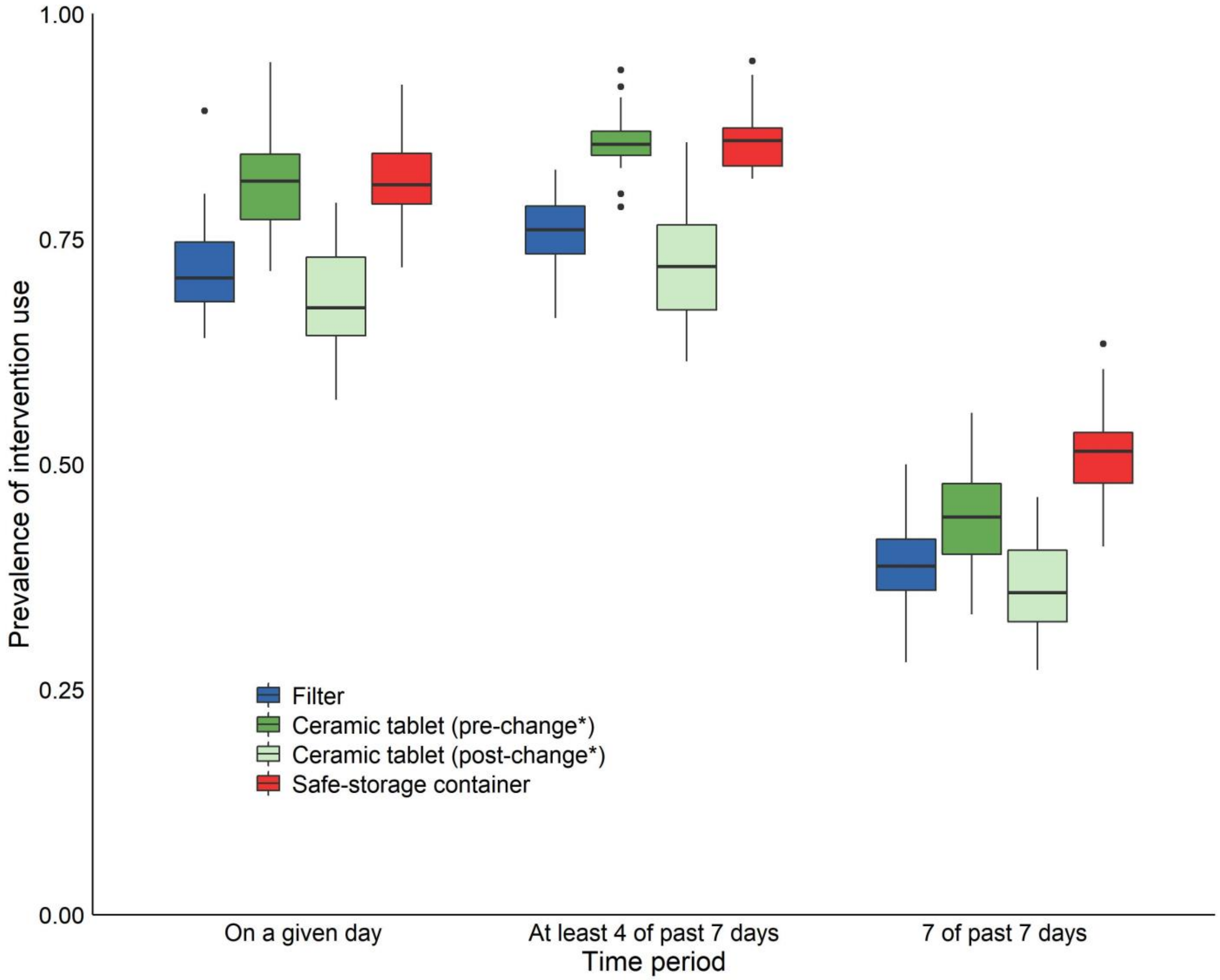

Figure S1. Objective intervention usage by intervention group as measured by the Smart Spout, a modified spigot that measured objective intervention usage based on the duration of each time the spigot was opened. Use on a given day was defined by the spigot being held open for at least 5 seconds, and results are reported for the prevalence of use on a given day, on at least 4 of the past 7 days, and on all 7 of the past 7 days.

*Pre-change refers to the time period before the interventions for households in the ceramic tablet group were replaced with ceramic filters. Post-change refers to the time period after the interventions were replaced with ceramic filters for this group.

Table S3. Baseline differences in anthropometry among 402 children with anthropometry at baseline.

Intervention	N	Mean (SD)	Difference* (95% CI)
HAZ			
Filter	99	-1.21 (1.20)	0.42 (0.08, 0.76)
Ceramic tablet	96	-1.17 (1.39)	0.46 (0.12, 0.80)
Safe-storage	99	-1.45 (1.14)	0.21 (-0.13, 0.55)
No intervention	89	-1.65 (1.37)	0.
WAZ			
Filter	98	-0.23 (1.08)	0.20 (-0.13, 0.53)
Ceramic tablet	96	-0.22 (1.18)	0.22 (-0.12, 0.55)
Safe-storage	101	-0.34 (1.14)	0.10 (-0.23, 0.43)
No intervention	96	-0.43 (1.35)	0.
WHZ			
Filter	95	0.65 (1.20)	0.11 (-0.27, 0.48)
Ceramic tablet	93	0.57 (1.41)	0.04 (-0.34, 0.41)
Safe-storage	96	0.68 (1.38)	0.12 (-0.26, 0.49)
No intervention	88	0.56 (1.42)	0.

*Adjusted for age using cubic splines with 4 knots

Table S4. Age-specific effects of water treatment interventions on child growth among 288 children that completed 24 months of follow-up and had a baseline anthropometric measure.

Baseline age	0-11 months			12-23 months			24-35 months		
Intervention	N children	ΔZ-score Mean (SD)	Mean ΔZ-score difference* (95% CI)	N children	ΔZ-score Mean (SD)	Mean ΔZ-score difference* (95% CI)	N children	ΔZ-score Mean (SD)	Mean ΔZ-score difference* (95% CI)
Height-for-age z-score									
Filter	30	-1.25 (1.14)	0.35 (-0.35, 1.06)	23	0.23 (0.74)	0.08 (-0.45, 0.61)	17	0.46 (0.68)	-0.19 (-0.76, 0.37)
Ceramic tablet	22	-1.18 (1.31)	0.40 (-0.36, 1.16)	27	-0.25 (1.10)	-0.24 (-0.75, 0.28)	20	0.64 (0.85)	0.03 (-0.49, 0.55)
Safe-storage	25	-0.99 (1.33)	0.57 (-0.17, 1.31)	32	0.13 (0.90)	0.05 (-0.44, 0.53)	18	0.33 (0.68)	-0.23 (-0.78, 0.31)
No intervention	19	-1.54 (1.05)	0.	23	0.08 (0.95)	0.	19	0.50 (1.01)	0.
Combined intervention	52	-1.22 (1.20)	0.05 (-0.45, 0.55)	50	-0.03 (0.98)	-0.11 (-0.46, 0.24)	37	0.56 (0.77)	0.05 (-0.33, 0.43)
Combined control	44	-1.23 (1.24)	0.	55	0.11 (0.91)	0.	37	0.42 (0.86)	0.
Weight-for-age z-score									
Filter	31	-0.34 (1.11)	-0.39 (-1.06, 0.27)	22	0.18 (0.57)	0.23 (-0.30, 0.76)	17	0.15 (1.04)	0.01 (-0.61, 0.62)
Ceramic tablet	24	0.02 (1.26)	-0.04 (-0.74, 0.66)	26	0.10 (1.01)	0.18 (-0.34, 0.69)	20	0.42 (0.71)	0.29 (-0.27, 0.85)
Safe-storage	26	0.01 (1.03)	-0.15 (-0.85, 0.55)	33	0.08 (1.00)	0.12 (-0.36, 0.60)	20	0.00 (0.72)	-0.05 (-0.62, 0.52)
No intervention	19	0.03 (1.32)	0.	23	-0.02 (0.76)	0.	20	0.07 (1.00)	0.
Combined intervention	55	-0.18 (1.18)	-0.15 (-0.61, 0.31)	48	0.14 (0.83)	0.13 (-0.21, 0.48)	37	0.29 (0.88)	0.20 (-0.21, 0.61)
Combined control	45	0.01 (1.15)	0.	56	0.04 (0.90)	0.	40	0.04 (0.86)	0.

Weight-for-height z-score									
Filter	30	0.37 (1.44)	-0.73 (-1.83, 0.37)	22	0.31 (0.90)	0.20 (-0.61, 1.01)	16	0.11 (1.66)	0.17 (-0.82, 1.17)
Ceramic tablet	22	0.71 (2.34)	-0.45 (-1.63, 0.74)	26	0.58 (1.41)	0.34 (-0.44, 1.12)	20	0.31 (1.42)	0.38 (-0.51, 1.27)
Safe-storage	24	0.43 (1.72)	-0.70 (-1.87, 0.46)	32	0.22 (1.40)	0.03 (-0.70, 0.77)	18	-0.23 (1.03)	-0.11 (-1.06, 0.83)
No intervention	18	0.99 (2.13)	0.	22	0.21 (1.44)	0.	19	-0.10 (1.46)	0.
Combined intervention	52	0.51 (1.86)	-0.22 (-0.99, 0.56)	48	0.45 (1.20)	0.25 (-0.27, 0.78)	36	0.22 (1.51)	0.35 (-0.31, 1.01)
Combined control	42	0.67 (1.90)	0.	54	0.22 (1.41)	0.	37	-0.17 (1.25)	0.

*Adjusted for age using cubic splines with 4 knots

Table S5. Intervention effects on stunting among 276 children that completed 24 months of follow-up and had a baseline height measured.

Intervention	N children	Stunted at baseline N (%)	Stunted at 24 months N (%)	Risk ratio (95% CI)
Filter	70	13 (18.6)	16 (22.9)	0.56 (0.30, 1.03)
Ceramic tablet	69	20 (29.0)	17 (24.6)	0.63 (0.34, 1.16)
Safe-storage	75	22 (29.3)	20 (26.7)	0.62 (0.35, 1.11)
No intervention	62	24 (38.7)	29 (46.8)	1.
Combined intervention	139	33 (23.7)	33 (23.7)	0.73 (0.46, 1.15)
Combined control	137	46 (33.6)	49 (35.8)	1.

*Adjusted for age using cubic splines with 4 knots and a quadratic term for baseline length-for-age z-score.

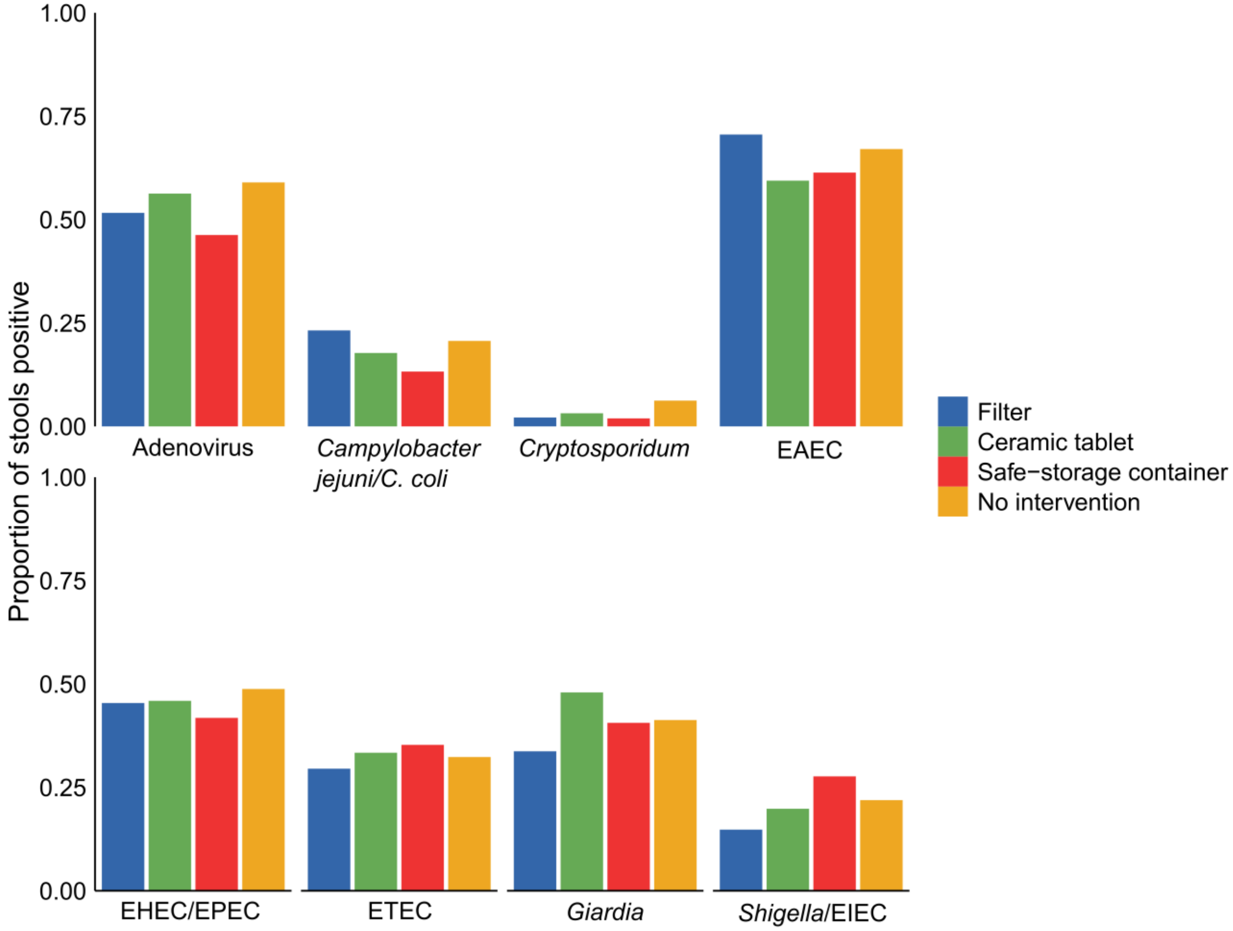

Figure S2. Enteric infection prevalence by intervention group at baseline among 394 children.

EAEC: enteroaggregative *E. coli*; EHEC: enterohemorrhagic *E. coli*; EPEC: enteropathogenic *E. coli*; ETEC: enterotoxigenic *E. coli*; EIEC: enteroinvasive *E. coli*

Table S6. Intention-to-treat intervention effects on prevalence of enteric pathogens at 6, 12, 18, and 24 months of follow-up among 365 children.

Pathogen	Intervention	N samples validly tested	Prevalence N (%)	Prevalence ratio* (95% CI)
EAEC	Filter	313	134 (42.8)	1.08 (0.91, 1.28)
	Ceramic tablet	298	134 (45.0)	1.17 (0.99, 1.38)
	Safe-storage	343	119 (34.7)	0.88 (0.72, 1.07)
	No intervention	273	105 (38.5)	1.
	Combined intervention	611	268 (43.9)	1.20 (1.06, 1.36)
	Combined control	616	224 (36.4)	1.
EHEC/EPEC	Filter	303	123 (40.6)	0.97 (0.79, 1.19)
	Ceramic tablet	289	131 (45.3)	1.10 (0.92, 1.33)
	Safe-storage	329	142 (43.2)	1.03 (0.86, 1.24)
	No intervention	260	107 (41.2)	1.
	Combined intervention	592	254 (42.9)	1.02 (0.89, 1.16)
	Combined control	589	249 (42.3)	1.
Giardia	Filter	313	90 (28.8)	0.80 (0.60, 1.06)
	Ceramic tablet	300	107 (35.7)	0.98 (0.78, 1.24)
	Safe-storage	344	105 (30.5)	0.86 (0.66, 1.11)
	No intervention	273	101 (37.0)	1.
	Combined intervention	613	197 (32.1)	0.96 (0.80, 1.16)
	Combined control	617	206 (33.4)	1.
C. jejuni/C. coli	Filter	308	22 (7.1)	1.15 (0.66, 1.99)
	Ceramic tablet	298	22 (7.4)	1.09 (0.62, 1.93)
	Safe-storage	341	17 (5.0)	0.76 (0.40, 1.45)
	No intervention	271	17 (6.3)	1.

	Combined intervention	606	44 (7.3)	1.29 (0.85, 1.98)
	Combined control	612	34 (5.6)	1.
Cryptosporidium	Filter	312	11 (3.5)	1.89 (0.69, 5.14)
	Ceramic tablet	299	10 (3.3)	1.81 (0.65, 5.03)
	Safe-storage	344	7 (2.0)	1.09 (0.36, 3.27)
	No intervention	271	5 (1.8)	1.
	Combined intervention	611	21 (3.4)	1.76 (0.90, 3.44)
	Combined control	615	12 (2.0)	1.
ETEC	Filter	296	49 (16.6)	0.90 (0.63, 1.29)
	Ceramic tablet	283	49 (17.3)	0.96 (0.69, 1.33)
	Safe-storage	321	45 (14.0)	0.86 (0.61, 1.22)
	No intervention	253	40 (15.8)	1.
	Combined intervention			1.01 (0.79, 1.29)
	Combined control			1.
Shigella/EIEC	Filter	312	50 (16.0)	1.31 (0.89, 1.91)
	Ceramic tablet	298	46 (15.4)	1.23 (0.83, 1.82)
	Safe-storage	343	41 (12.0)	0.98 (0.67, 1.45)
	No intervention	271	33 (12.2)	1.
	Combined intervention	610	96 (15.7)	1.28 (0.98, 1.67)
	Combined control	614	74 (12.1)	1.
Adenovirus	Filter	291	95 (32.6)	1.28 (0.97, 1.71)
	Ceramic tablet	276	90 (32.6)	1.29 (0.99, 1.68)
	Safe-storage	322	95 (29.5)	1.21 (0.92, 1.60)
	No intervention	251	61 (24.3)	1.
	Combined intervention	567	185 (32.6)	1.15 (0.97, 1.36)

	Combined control	573	156 (27.2)	1.
Total number of pathogens detected†	Filter	313	2 (1, 3)	1.06 (0.93, 1.20)
	Ceramic tablet	300	2 (1, 3)	1.13 (1.00, 1.28)
	Safe-storage	344	1 (1, 3)	0.97 (0.85, 1.10)
	No intervention	273	1 (1, 2)	1.
	Combined intervention	613	2 (1, 3)	1.11 (1.02, 1.21)
	Combined control	617	1 (1, 2)	1.

*Adjusted for age and year of stool testing.
†Effects estimated are detection rate ratios.
EAEC: enteroaggregative *E. coli*; EHEC: enterohemorrhagic *E. coli*; EPEC: enteropathogenic *E. coli*; ETEC: enterotoxigenic *E. coli*; EIEC: enteroinvasive *E. coli*

Table S7. As-treated intervention effects on prevalence of enteric pathogens at 6, 12, 18, and 24 months of follow-up among 365 children.

Pathogen	Intervention	N samples validly tested	Prevalence N (%)	Prevalence ratio* (95% CI)
EAEC	Filter	186	88 (47.3)	1.03 (0.85, 1.25)
	No silver filter	276	103 (37.3)	1.28 (0.93, 1.75)
	Ceramic tablet	149	77 (51.7)	1.12 (0.94, 1.33)
	Safe-storage	343	119 (34.7)	0.88 (0.72, 1.07)
	No intervention	273	105 (38.5)	1.
	Combined intervention[†]	335	165 (49.3)	1.15 (1.00, 1.32)
	Combined control	616	224 (36.4)	1.
EHEC/EPEC	Filter	176	79 (44.9)	1.00 (0.79, 1.25)
	No silver filter	278	106 (38.1)	1.09 (0.82, 1.45)
	Ceramic tablet	138	69 (50.0)	1.11 (0.90, 1.38)
	Safe-storage	329	142 (43.2)	1.04 (0.86, 1.25)
	No intervention	260	107 (41.2)	1.
	Combined intervention[†]	314	148 (47.1)	1.03 (0.88, 1.20)
	Combined control	589	249 (42.3)	1.
Giardia	Filter	186	52 (28.0)	0.78 (0.58, 1.05)
	No silver filter	278	103 (37.1)	0.99 (0.75, 1.31)
	Ceramic tablet	149	42 (28.2)	0.75 (0.54, 1.04)
	Safe-storage	344	105 (30.5)	0.85 (0.66, 1.10)
	No intervention	273	101 (37.0)	1.
	Combined intervention[†]	335	94 (28.1)	0.84 (0.67, 1.05)
	Combined control	617	206 (33.4)	1.
C. jejuni/C. coli	Filter	181	17 (9.4)	1.11 (0.63, 1.94)

	No silver filter	278	12 (4.3)	--[‡]
	Ceramic tablet	147	15 (10.2)	0.99 (0.53, 1.84)
	Safe-storage	341	17 (5.0)	0.76 (0.40, 1.45)
	No intervention	271	17 (6.3)	1.
	Combined intervention[†]	328	32 (9.8)	1.21 (0.79, 1.85)
	Combined control	612	34 (5.6)	1.
Cryptosporidium	Filter	185	7 (3.8)	1.73 (0.60, 5.00)
	No silver filter	278	11 (4.0)	--[‡]
	Ceramic tablet	148	3 (2.0)	0.90 (0.23, 3.57)
	Safe-storage	344	7 (2.0)	1.07 (0.35, 3.20)
	No intervention	271	5 (1.8)	1.
	Combined intervention[†]	333	10 (3.0)	1.33 (0.63, 2.80)
	Combined control	615	12 (2.0)	1.
ETEC	Filter	169	31 (18.3)	0.76 (0.52, 1.13)
	No silver filter	278	32 (11.5)	--[‡]
	Ceramic tablet	132	35 (26.5)	0.92 (0.65, 1.31)
	Safe-storage	321	45 (14.0)	0.86 (0.61, 1.22)
	No intervention	253	40 (15.8)	1.
	Combined intervention[†]	301	66 (21.9)	0.91 (0.70, 1.19)
	Combined control	574	85 (14.8)	1.
Shigella/EIEC	Filter	185	32 (17.3)	1.26 (0.82, 1.93)
	No silver filter	278	36 (12.9)	1.23 (0.71, 2.13)
	Ceramic tablet	147	28 (19.0)	1.28 (0.79, 2.06)
	Safe-storage	343	41 (12.0)	0.98 (0.67, 1.45)
	No intervention	271	33 (12.2)	1.

	Combined intervention†	332	60 (18.1)	1.28 (0.98, 1.67)
	Combined control	614	74 (12.1)	1.
Adenovirus	Filter	184	66 (35.9)	1.30 (0.96, 1.76)
	No silver filter	235	63 (26.8)	1.28 (0.84, 1.96)
	Ceramic tablet	148	56 (37.8)	1.32 (0.98, 1.76)
	Safe-storage	322	95 (29.5)	1.22 (0.93, 1.61)
	No intervention	251	61 (24.3)	1.
	Combined intervention†	332	122 (36.7)	1.16 (0.95, 1.42)
	Combined control	573	156 (27.2)	1.
Total number of pathogens detected§	Filter	186	2 (1, 3)	1.02 (0.89, 1.18)
	No silver filter	278	2 (1, 2)	1.18 (0.99, 1.40)
	Ceramic tablet	149	2 (1, 3)	1.05 (0.90, 1.21)
	Safe-storage	344	1 (1, 3)	0.97 (0.85, 1.10)
	No intervention	273	1 (1, 2)	1.
	Combined intervention†	335	2 (1, 3)	1.05 (0.95, 1.17)
	Combined control	617	1 (1, 2)	1.

*Adjusted for age and year of stool testing.
†Excludes no silver filter group.
‡Model did not converge.
§Effects estimated are detection rate ratios.

EAEC: enteroaggregative *E. coli*; EHEC: enterohemorrhagic *E. coli*; EPEC: enteropathogenic *E. coli*; ETEC: enterotoxigenic *E. coli*; EIEC: enteroinvasive *E. coli*

Table S8. Intention-to-treat intervention effects on prevalence of enteric pathogens at 6, 12, 18, and 24 months of follow-up among 349 children, excluding samples tested in 2016 and 2017.

Pathogen	Intervention	N samples validly tested	Prevalence N (%)	Prevalence ratio* (95% CI)
EAEC	Filter	247	89 (36.0)	1.16 (0.91, 1.40)
	Ceramic tablet	231	81 (35.1)	1.15 (0.89, 1.50)
	Safe-storage	277	86 (31.0)	1.02 (0.78, 1.33)
	No intervention	215	65 (30.2)	1.
	Combined intervention	202	67 (33.2)	1.14 (0.96, 1.36)
	Combined control	492	151 (30.7)	1.
EHEC/EPEC	Filter	247	91 (36.8)	0.98 (0.76, 1.25)
	Ceramic tablet	233	93 (39.9)	1.07 (0.84, 1.35)
	Safe-storage	278	118 (42.4)	1.14 (0.91, 1.41)
	No intervention	215	80 (37.2)	1.
	Combined intervention	202	78 (38.6)	0.95 (0.81, 1.11)
	Combined control	493	198 (40.2)	1.
Giardia	Filter	269	70 (26.0)	0.77 (0.56, 1.06)
	Ceramic tablet	253	92 (36.4)	1.09 (0.85, 1.39)
	Safe-storage	294	89 (30.3)	0.96 (0.79, 1.17)
	No intervention	230	80 (34.8)	1.
	Combined intervention	244	59 (24.2)	0.96 (0.79, 1.17)
	Combined control	524	169 (32.3)	1.
C. jejuni/C. coli	Filter	269	15 (5.6)	1.20 (0.56, 2.58)
	Ceramic tablet	253	14 (5.5)	1.19 (0.54, 2.63)
	Safe-storage	294	13 (4.4)	0.95 (0.43, 2.08)
	No intervention	230	10 (4.3)	1.

	Combined intervention	244	17 (7.0)	1.23 (0.72, 2.10)
	Combined control	524	23 (4.4)	1.
Cryptosporidium	Filter	269	10 (3.7)	2.80 (0.80, 9.77)
	Ceramic tablet	253	9 (3.6)	2.70 (0.76, 9.58)
	Safe-storage	294	6 (2.0)	1.51 (0.39, 5.85)
	No intervention	230	3 (1.3)	1.
	Combined intervention	244	8 (3.3)	2.14 (1.01, 4.56)
	Combined control	524	9 (1.7)	1.
ETEC	Filter	240	28 (11.7)	1.30 (0.75, 2.27)
	Ceramic tablet	226	23 (10.2)	1.17 (0.66, 2.10)
	Safe-storage	269	23 (8.6)	0.96 (0.53, 1.75)
	No intervention	209	18 (8.6)	1.
	Combined intervention	188	19 (10.1)	1.24 (0.84, 1.84)
	Combined control	478	41 (8.6)	1.
Shigella/EIEC	Filter	240	30 (12.5)	1.17 (0.72, 1.87)
	Ceramic tablet	226	33 (14.6)	1.37 (0.84, 2.25)
	Safe-storage	269	25 (9.3)	0.87 (0.52, 1.46)
	No intervention	209	22 (10.5)	1.
	Combined intervention	188	27 (14.4)	1.37 (0.97, 1.92)
	Combined control	478	47 (9.8)	1.
Adenovirus	Filter	220	61 (27.7)	1.23 (0.84, 1.81)
	Ceramic tablet	203	66 (32.5)	1.47 (1.03, 2.10)
	Safe-storage	247	64 (25.9)	1.15 (0.80, 1.66)
	No intervention	189	42 (22.2)	1.
	Combined intervention	188	64 (34.0)	1.17 (0.93, 1.47)

	Combined control	436	106 (24.3)	1.
Total number of pathogens detected†	Filter	247	1 (1, 2)	1.06 (0.91, 1.23)
	Ceramic tablet	233	2 (1, 2)	1.19 (1.02, 1.37)
	Safe-storage	278	1 (1, 2)	1.03 (0.89, 1.20)
	No intervention	215	1 (1, 2)	1.
	Combined intervention	480	1 (1, 2)	1.10 (0.99, 1.22)
	Combined control	493	1 (1, 2)	1.

*Adjusted for age and year of stool testing.
†Effects estimated are detection rate ratios.
EAEC: enteroaggregative *E. coli*; EHEC: enterohemorrhagic *E. coli*; EPEC: enteropathogenic *E. coli*; ETEC: enterotoxigenic *E. coli*; EIEC: enteroinvasive *E. coli*
